# Efficacy of broccoli and glucoraphanin in COVID-19: From hypothesis to proof-of-concept with three experimental clinical cases

**DOI:** 10.1016/j.waojou.2020.100498

**Published:** 2020-12-09

**Authors:** Jean Bousquet, Vincent Le Moing, Hubert Blain, Wienczyslawa Czarlewski, Torsten Zuberbier, Rafael de la Torre, Nieves Pizarro Lozano, Jacques Reynes, Anna Bedbrook, Jean-Paul Cristol, Alvaro A. Cruz, Alessandro Fiocchi, Tari Haahtela, Guido Iaccarino, Ludger Klimek, Piotr Kuna, Erik Melén, Joaquim Mullol, Boleslaw Samolinski, Arunas Valiulis, Josep M. Anto

**Affiliations:** a Charité, Universitätsmedizin Berlin, Humboldt-Universität zu Berlin, and Berlin Institute of Health, Comprehensive Allergy Center, Department of Dermatology and Allergy, Berlin, Germany; b MACVIA France, University Hospital, Montpellier, France; c Maladies Infectiouses et Tropicales, CHU Montpellier, France; d Department of Geriatrics, Montpellier University Hospital, Montpellier, France; e Medical Consulting Czarlewski, Levallois, France; f MASK-air, Montpellier, France; g Laboratoire de Biochimie et Hormonologie, PhyMedExp, Université de Montpellier, INSERM, CNRS, CHU de Montpellier, France; h CIBER Fisiopatologia de La Obesidad y Nutrición (CIBEROBN), Madrid, Spain; i IMIM (Hospital del Mar Research Institute), Barcelona, Spain; j Universitat Pompeu Fabra (UPF), Barcelona, Spain; k CIBER Epidemiología y Salud Pública (CIBERESP), Barcelona, Spain; l ISGlobal. ISGlobAL, Barcelona, Centre for Research in Environmental Epidemiology (CREAL), Barcelona, Spain; m Fundação ProAR, Federal University of Bahia and GARD/WHO Planning Group, Salvador, Brazil; n Division of Allergy, Department of Pediatric Medicine - The Bambino Gesù Children's Research Hospital Holy see, Rome, Italy; o Skin and Allergy Hospital, Helsinki University Hospital, And University of Helsinki, Helsinki, Finland; p Department of Advanced Biomedical Sciences, Federico II University, Napoli, Italy; q Center for Rhinology and Allergology, Wiesbaden, Germany; r Division of Internal Medicine, Asthma and Allergy, Barlicki University Hospital, Medical University of Lodz, Poland; s Institute of Environmental Medicine, Karolinska Institutet and Sachs' Children's Hospital, Stockholm, Sweden; t Rhinology Unit & Smell Clinic, ENT Department, Hospital Clinic - Clinical & Experimental Respiratory Immunoallergy, IDIBAPS, CIBERES, Universitat de Barcelona, Barcelona, Spain; u Department of Prevention of Environmental Hazards and Allergology, Medical University of Warsaw, Poland; v Vilnius University Faculty of Medicine, Institute of Clinical Medicine & Institute of Health Sciences, Vilnius, Lithuania

**Keywords:** COVID-19, Nrf2, Broccoli, Cough challenge, TRPA1, TRPV1, ACE, Angiotensin converting enzyme, AT_1_R, Angiotensin II receptor type 1, BMI, Body mass index, Broccoli, Broccoli seed capsules, COVID-19, Coronavirus 19 disease, NAPQI, N-acetyl-p-benzoquinone imine, Nrf2, Nuclear factor (erythroid-derived 2)-like 2, SARS, Severe acute respiratory syndrome, SARS-Cov-2, Severe acute respiratory syndrome coronavirus 2, TRP, Transient receptor potential, TRPA1, Transient receptor potential ankyrin 1, TRPV1, Transient receptor potential vanillin 1, VAS, Visual analogue scale

## Abstract

COVID-19 is described in a clinical case involving a patient who proposed the hypothesis that Nuclear factor (erythroid-derived 2)-like 2 (Nrf2)-interacting nutrients may help to prevent severe COVID-19 symptoms. Capsules of broccoli seeds containing glucoraphanin were being taken before the onset of SARS-CoV-2 infection and were continued daily for over a month after the first COVID-19 symptoms. They were found to reduce many of the symptoms rapidly and for a duration of 6–12 h by repeated dosing. When the patient was stable but still suffering from cough and nasal obstruction when not taking the broccoli capsules, a double-blind induced cough challenge confirmed the speed of onset of the capsules (less than 10 min). A second clinical case with lower broccoli doses carried out during the cytokine storm confirmed the clinical benefits already observed. A third clinical case showed similar effects at the onset of symptoms. In the first clinical trial, we used a dose of under 600 μmol per day of glucoraphanin. However, such a high dose may induce pharmacologic effects that require careful examination before the performance of any study. It is likely that the fast onset of action is mediated through the TRPA1 channel. These experimental clinical cases represent a proof-of-concept confirming the hypothesis that Nrf2-interacting nutrients are effective in COVID-19. However, this cannot be used in practice before the availability of further safety data, and confirmation is necessary through proper trials on efficacy and safety.

## Introduction

The present report consists of the self-description of a COVID-19 case where the patient – who is also the author — describes his clinical course as well as the anecdotal evidence of symptom improvement after treatment with broccoli capsules. Since the patient himself developed the concept that Nrf2-interacting nutrients may help to prevent severe COVID-19 symptoms or, less likely, to prevent SARS-CoV-2 infection,^1^, ^2^, ^3^ he took low-dose broccoli capsules with glucoraphanin and myrosinase to prevent the infection, and higher doses when COVID-19 was clinically evident. Broccoli was chosen as it contains glucoraphanin that is obtained from cruciferous vegetables such as broccoli, Brussels sprouts, and cabbage. The most potent Nrf2 natural activator is sulforaphane,^4^, ^5^, ^6^, ^7^, ^8^, ^9^, ^10^ but it is difficult to deliver in an enriched and stable form for purposes of direct human consumption.^11^ Thus, glucoraphanin, the precursor of sulforaphane, is administered orally with myrosinase, the enzyme that transforms glucoraphanin into sulforaphane.

After the cytokine storm, the patient continued to suffer from cough and nasal obstruction when he was not taking the broccoli capsules. A double-blind induced cough challenge was carried out to assess the speed of onset of the broccoli capsules.

### Hypothesis: the heterogeneity of COVID-19 death rates between countries could be partially due to the consumption of Nrf2-interacting nutrients

There are large between- and within-country variations in COVID-19 death rates. Some very low death rate settings — such as those of Eastern Asia, Central Europe, the Balkans and Africa — have a common feature of eating large quantities of fermented foods and some specific vegetables such as cabbage. Although biases exist when examining ecological studies, fermented vegetables and cabbage can be associated with lower death rates in European countries. Many foods have antioxidant properties and many mechanisms may be involved. However, the activation of the Nrf2 (Nuclear factor (erythroid-derived 2)-like 2) anti-oxidant transcription factor may be of primary importance. Nrf2 is the main regulator of the antioxidant response in humans. It modulates the expression of hundreds of genes, including not only the antioxidant enzymes (ie, glutathione related), but also the large number of genes that control seemingly disparate physiopathological processes.^12^^,^^13^ Nrf2 activity can block the Angiotensin II receptor type 1 (AT_1_R) axis as well as endoplasmic reticulum stress.

Cabbage contains precursors of sulforaphane, the most active natural activator of Nrf2. Fermented vegetables contain many lactobacilli, which are also potent Nrf2 activators. Three examples where the association with COVID-19 mortality could have been influenced by the presence or lack of fermented vegetable consumption were proposed: Kimchi in Korea, westernized foods, and the slum paradox. It was expected that the COVID-19 pandemic would be catastrophic in the slum areas of middle- or low-income countries due to inequalities in the workforce of essential services, poverty, access to care, or air pollution.^14^^,^^15^ In Mumbai, although a large number of people living in slums are infected by COVID-19, the death rate is very low.^16^ It is possible that eating fermented foods and spices may prevent against severe COVID-19. Kimchi is a traditional Korean fermented food usually containing cabbage and/or radish (Nrf2),^3^^,^^17^ garlic, red pepper, ginger, and other spices (TRPA1/TRPV1).^17^ Fermentation by lactic acid bacilli increases the health benefits of Kimchi (Nrf2). It is proposed that Kimchi and other fermented vegetables and spices largely eaten in Asia may partly explain the low prevalence of severe COVID-19 in these countries.^3^

There are many Nrf2-interacting nutrients (berberine, curcumin, epigallocatechin gallate, genistein, quercetin, resveratrol, sulforaphane, and many others) that all act similarly to reduce insulin resistance, endothelial damage, lung injury, and cytokine storm (Bousquet et al, submitted). They also act on the same mechanisms (mTOR: Mammalian target of rapamycin, PPARγ: Peroxisome proliferator-activated receptor, NFκB: Nuclear factor kappa B, ERK: Extracellular signal-regulated kinases and eIF2α: Elongation initiation factor 2α). As a result, they may be important in mitigating the severity of COVID-19, acting through the endoplasmic reticulum stress or ACE-Angiotensin–II–AT_1_R axis (AT_1_R) pathway. Interestingly, geographical areas with very low COVID-19 mortality are those with the lowest prevalence of obesity (Sub-Saharan Africa and Asia).

We proposed that Nrf2-interacting foods and nutrients can re-balance insulin resistance and have a significant effect on COVID-19 severity. It is possible, therefore, that the intake of these foods may restore an optimal natural balance for the Nrf2 pathway and be of interest in the mitigation of COVID-19 severity. Understanding the balance between Nrf2-interacting foods and nutrients would help to: (i) better understand the mechanisms of the oxidative stress in insulin resistance-associated diseases, (ii) develop optimal Nrf2-interacting nutrients and diets to reduce the prevalence and severity of IR diseases, (iii) optimize Nrf2 drug development, and (iv) develop these strategies to mitigate COVID-19 severity.

### Fast onset of broccoli capsules with glucoraphanin on COVID-19 symptoms

#### Clinical case 1

The hypothesis that an oral administration of broccoli seeds and glucoraphanin capsules (from now on broccoli capsules) could beneficially ameliorate the COVID-19 course was tested on a 73-year old man, a former professor of respiratory medicine at the Montpellier University of France (BMI 23, allergic rhinitis, intermittent untreated asthma, and well-controlled type-2 diabetes under metformin, HbA1C: 6.2%). Before developing COVID-19, the patient self-prescribed broccoli capsules OD in the morning for 45 days (Aerobiane, Pileje, France: broccoli seeds 300 mg + glucoraphanin 30 mg, and myrosinase) in the hope to prevent the onset of COVID-19. But he did contract the infection. After mass spectrometry analysis, it was found that the dose of glucoraphanin was 9 mg, around 25 μmol.

**Fig. 1:**
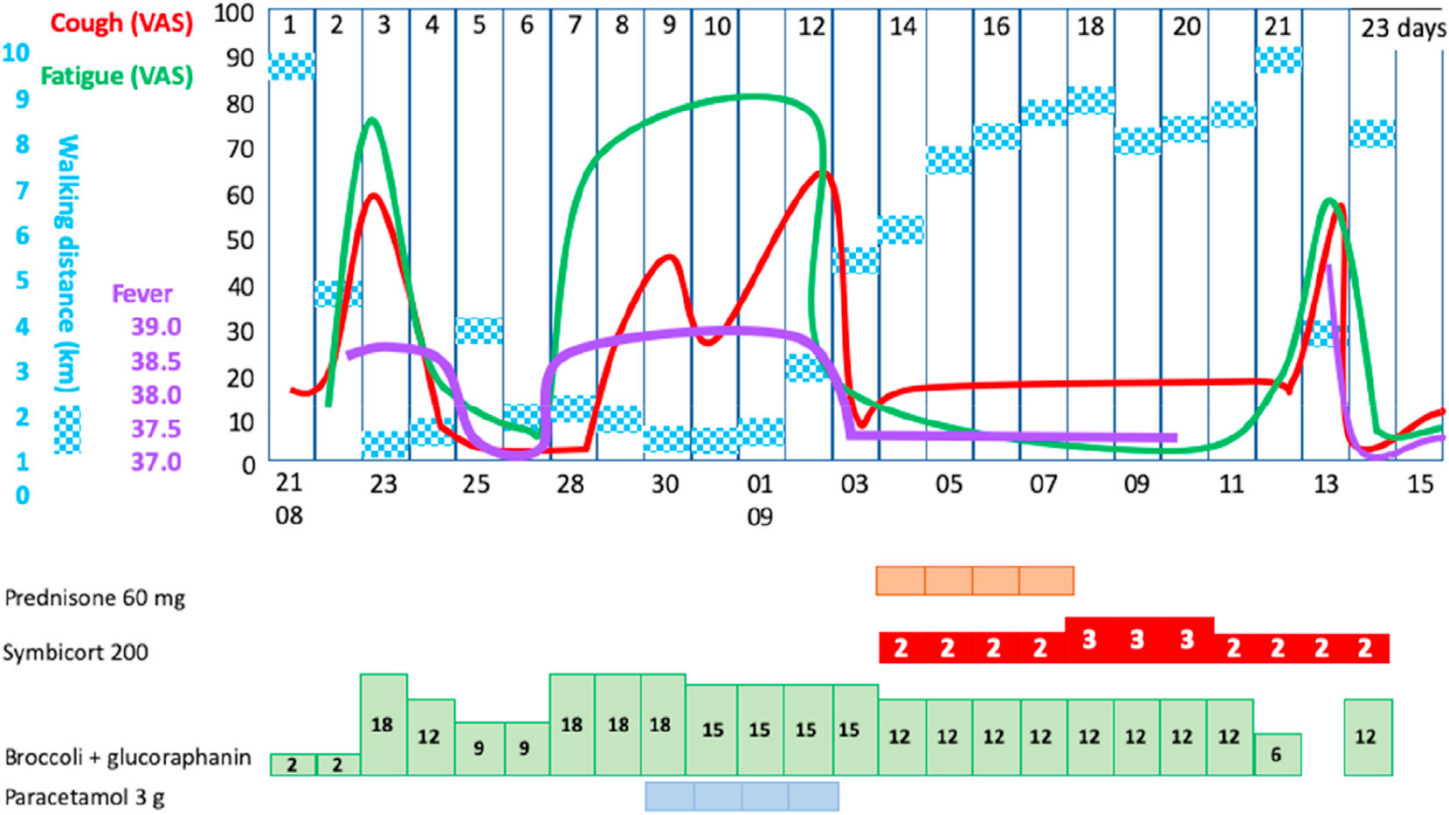
Clinical case. Only The highest daily score of symptoms for the day are shown. “Days” indicate the number of days since the first symptoms. Fatigue VAS: 0: none, 10: extreme

**Day 1:** He started to experience COVID-19 symptoms on August 22, 2020 (Fig. 1). A few months ago, the patient developed a COVID-19 app (MASK-COVID) — available on Android — using the estimation of symptom severity by visual analogue scale (VAS) based on his previous experience of developing an allergy app.^18^ He used VAS to score his daily symptoms on paper, as the app is not yet available on iOS and the patient did not have access to Android. For each day, the maximal VAS was reported. For **days 1 to 3**, VAS was estimated retrospectively.

**Days 2**–**4:** The symptoms began with mild cough (VAS: 2/10), followed 12 h later by severe fatigue (VAS: 6/10) and then, at 24 h, fever (38.9°). At 36 h, he also experienced loss of appetite (VAS: 7/10), nausea (VAS: 3/10), diarrhea (VAS: 2/10), hyposmia (VAS: 3/10), dysgeusia (VAS: 3/10), and nasal obstruction (VAS: 4/10) (Fig. 1).

**Fig. 2:**
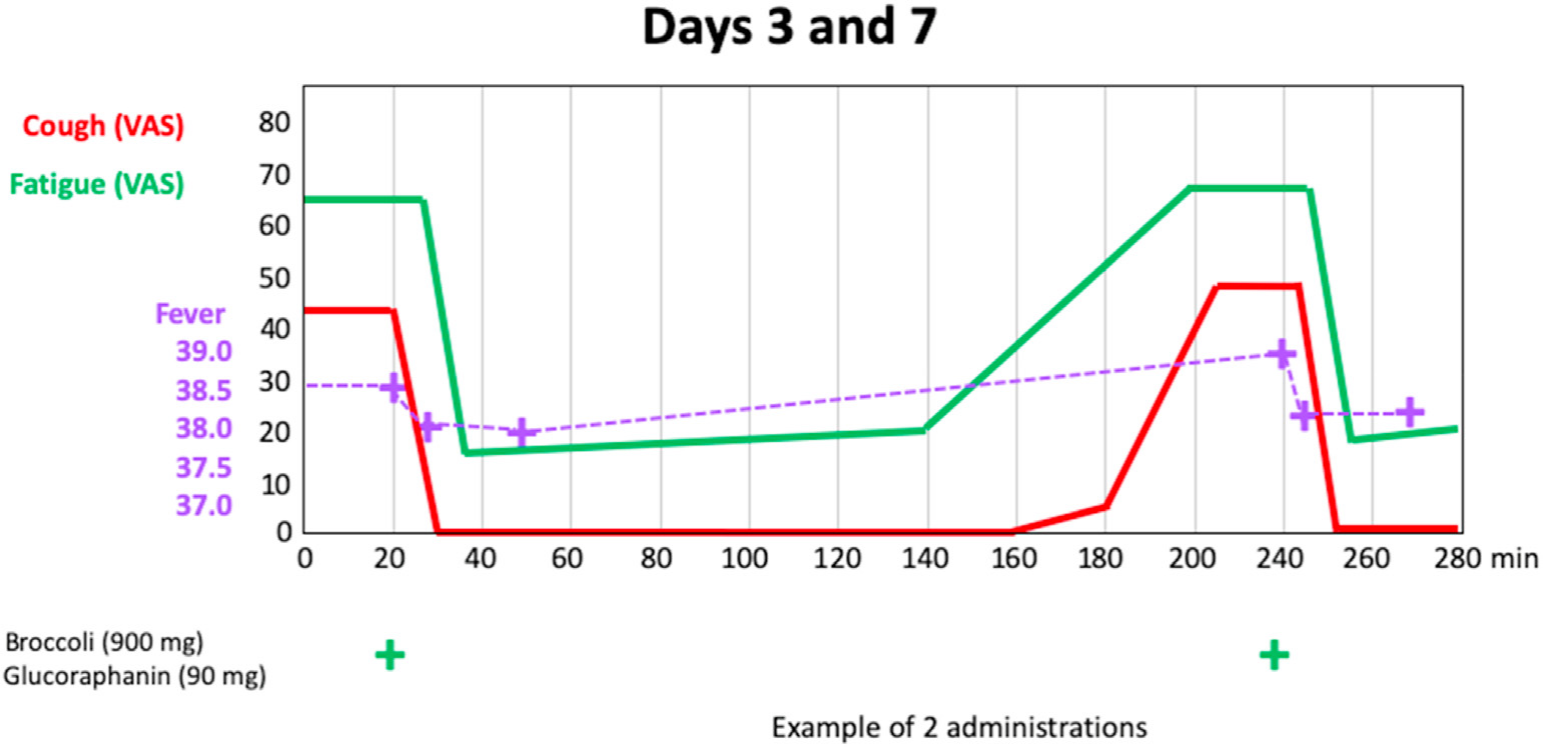
Evolution of symptoms after ingestion of broccoli capsules. VAS: 0: none, 10 extreme

The patient increased the dosage of the broccoli capsules on day 3. The first dose of 600 mg of broccoli (4 capsules) (7:00) improved the symptoms incompletely, and he therefore took a higher dose 5 h later (900 mg). Interestingly, the ingestion of the capsules always induced the same effect with rapid disappearance of cough, nasal obstruction, nausea and diarrhea, improvement in fatigue, and some reduction in fever (Fig. 2). He repeated this dose when nasal obstruction reappeared and cough increased in severity (VAS>5/10), needing capsules every 6–8 h (12:00, 18:00, 23:00, 7:00, 13:00) to achieve symptom control (Fig. 2). At 14:30 on day 4, the patient felt well, had no fever, and was asymptomatic.

Since the patient found the symptoms bothersome on day 3, he contacted Prof. H. Blain (HB: geriatrician, Montpellier) — with whom he is working on COVID-19 in home care services^19^ — and discussed the Nrf2 hypothesis.^2^^,^^3^ HB considered that the initial symptoms were severe and contacted (day 4) Prof. V. Le Moing (VLM: Director of the COVID-19 clinic of the Montpellier hospital). Then, for the next 3 weeks, the patient had daily contact with VLM by phone or SMS to adjust the treatment. Hospitalization would have been proposed at the very first sign of severity of the cytokine storm (eg, respiratory rate >24/min). To optimize shared decision making, the patient scored all of the symptoms on a VAS scale (0–10) and reported them at least once daily to VLM. He also had regular contact with Dr. W Czarlewski with whom he developed the Nrf2 hypothesis.^2^^,^^3^ Dr. Czarlewski is knowledgeable on natural medicine and was able to discuss the dosage of the broccoli capsules.

**Days 5**–**6:** The patient took 3 broccoli capsules (450 mg) 3 times per day and felt well.

**Days 7**–**12** (Fig. 1)**:** The symptoms of the cytokine storm began on day 7, and the patient increased the dose of broccoli (900 mg). He noted the same rapid effect for the following 2 days (days 7 and 8) (Fig. 2) and took 1 dose every 6 h when cough reappeared and increased in severity (VAS>5). The total daily dose of broccoli seeds was 2.7 g and of glucoraphanin 270 mg (around 600 μmol).

On **day 8**, fever did not decrease and so he added paracetamol (3 g a day for 4 days). Fever reduced to 36.9° (morning) and 37.3° (evening) and remained the same up to day 13. Interestingly, the intervals between the broccoli doses (900 mg) could then be increased to 10–12 h. The patient's respiratory rate was never >20/min. Since the symptoms were controlled (including fatigue VAS: 2–4/10) and the respiratory rate was not increased, the patient stayed at home, under the twice-daily supervision of VLM.

**Days 13**–**20:** On day 13, the patient felt well and had no fever. A dose of 900 mg of broccoli BID was ingested. However, he developed a productive cough (yellow eosinophil-like sputum). Further to shared decision making with VLM, the patient took Prednisone (60 mg/day) on days 14–17 which resulted in translucent sputum. After day 22, the sputum production ceased.

**Fig. 3:**
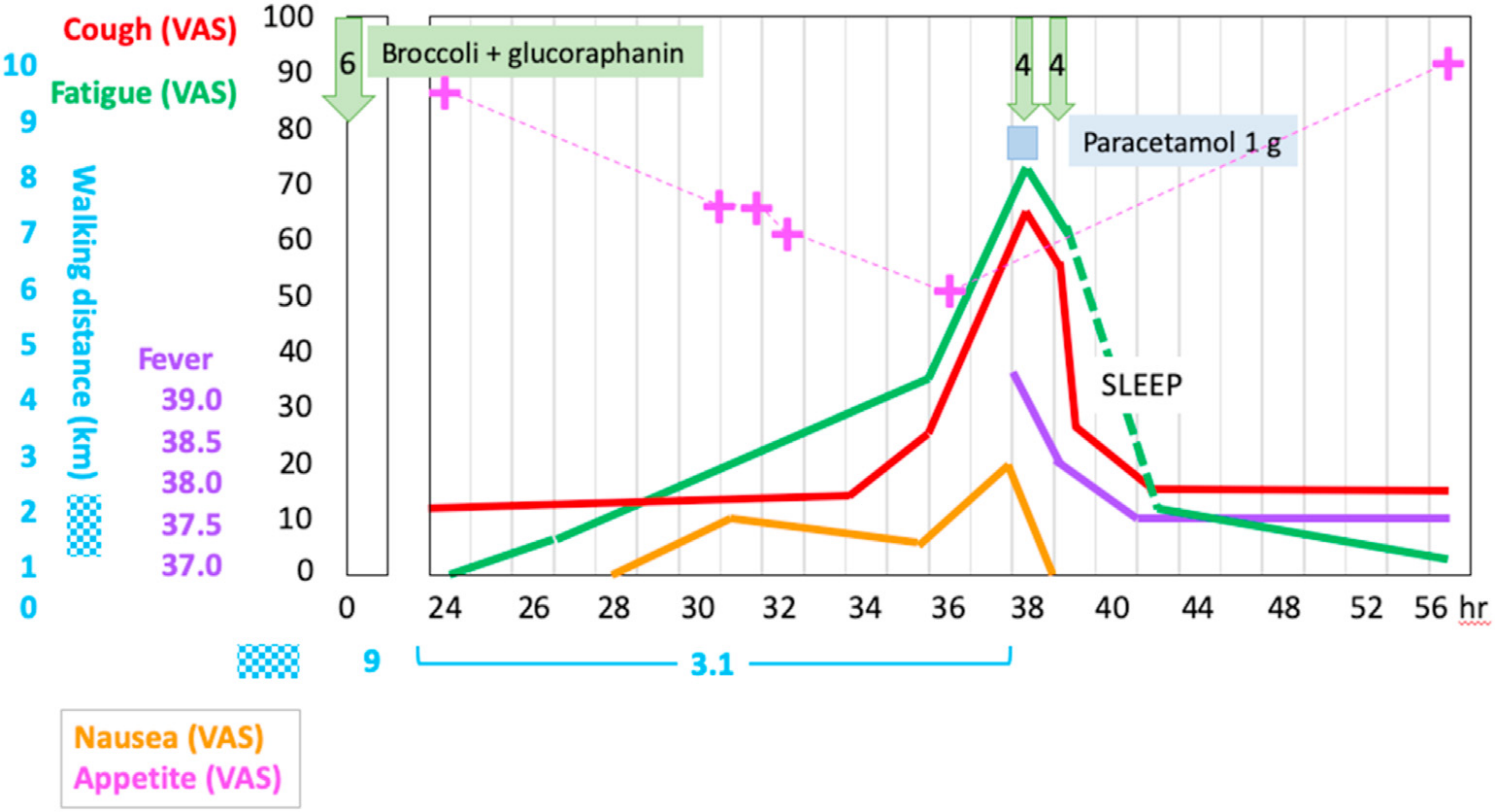
Evolution of symptoms at days 21–22. Fatigue VAS: 0: none, 10 extreme, for appetite: loss of appetite

**Days 21**–**24:** On day 21, the patient stopped the broccoli capsules because he was travelling and had forgotten them (Fig. 3). He experienced a recurrence of symptoms with increasing fatigue, loss of appetite, nausea and, later, within 24 h, cough and fever. He was obliged to wait for 3h after the onset of symptoms before taking the capsules and had not experienced such a severe cough since the beginning of the infection. The patient took 600 mg of broccoli capsules and, within 5 min, the cough had reduced and the nausea had disappeared. Given that his fever was still at 38.9°, he took 1 g of paracetamol. The fever decreased to 38.1° within minutes. Since cough was not reduced, he took another 600 mg of broccoli capsules and it improved. After 2 h of perspiring, the patient's temperature had fallen to 36.9°. He then felt perfectly well and experienced no further fatigue or cough. The next morning, the patient felt well with a return of appetite and no fatigue. He then took 300 mg of broccoli morning and evening (days 23–24).

**Fig. 4:**
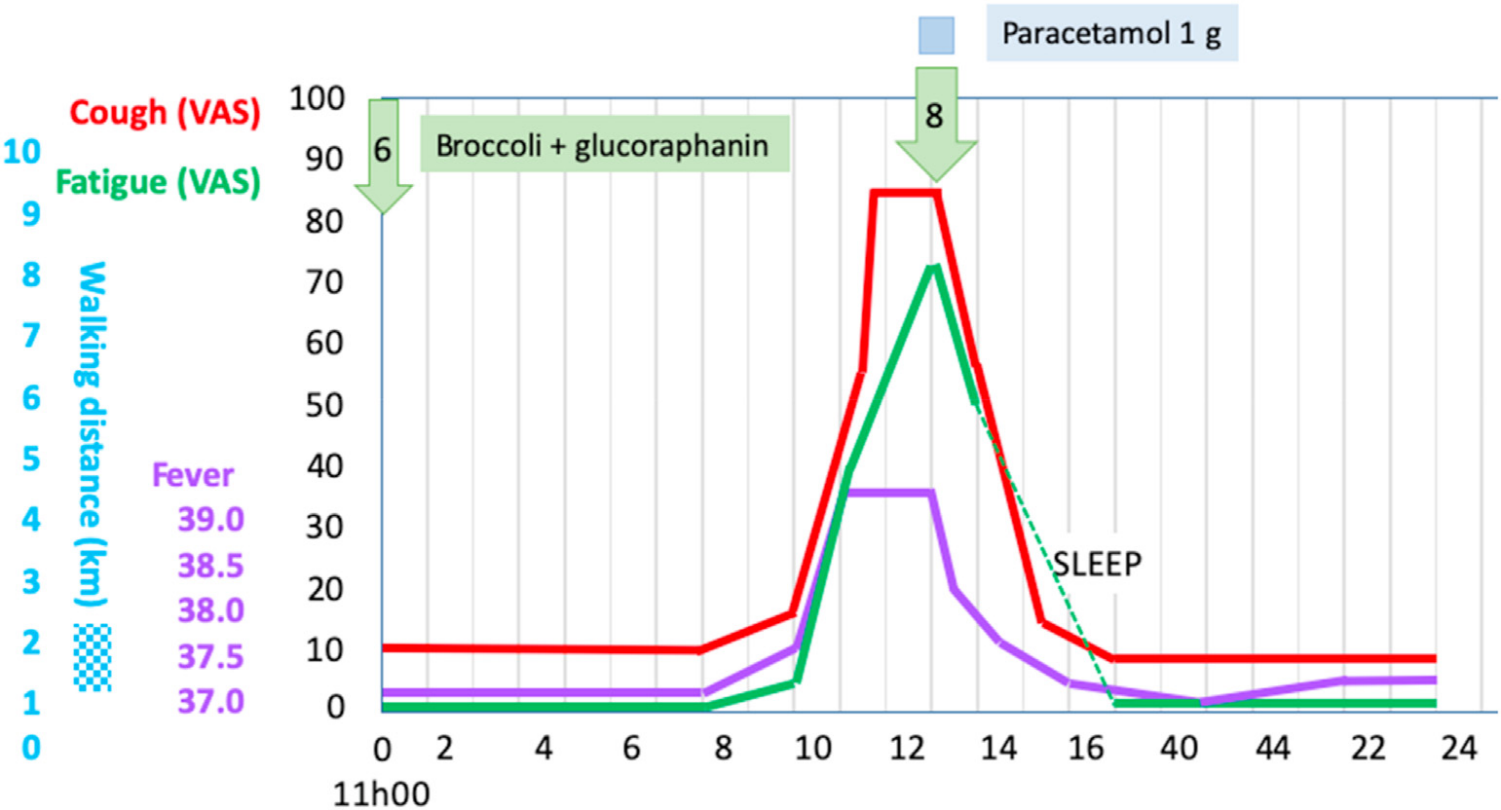
Evolution of symptoms at day 25

**Day 25:** The patient purposely delayed the broccoli treatment to determine whether it was needed, and the same symptoms returned (fatigue, cough, nausea, fever) (Fig. 4). He then decided to wait for 2 h before taking the broccoli capsules. The cough became very severe (VAS 8/10) and his temperature increased to 38.9°. He took the broccoli capsules (1200 mg) and paracetamol, and the same sequence of events was observed.

**Days 26**–**38:** Every day, the patient took 2 capsules (300 mg) in the morning and 2 during the day. After ingestion of the broccoli, he was rapidly almost symptom free (VAS 1–2 for nasal symptoms and spontaneous cough). After 10–12 h, the cough and nasal obstruction returned. When cough VAS was over 5, he took the broccoli capsules. On days 30 and 31, the patient did not take the capsules and did a double-blind, placebo-controlled challenge.

Over the course of the disease, the patient attempted to improve cough using formoterol-budesonide low dose, but there was no effect. He also attempted to control nasal obstruction using azelastine-fluticasone propionate without any effect.

##### Biological tests and CT-scan

The patient had a first positive PCR test to SARS-CoV-2 on August 24 (day 3). The test was repeated on September 15 and was still positive, showing IgG antibodies to SARS-CoV-2. He had a moderate inflammation (September 15): CRP 30 mg/l, D-dimers (945 ng/ml), no lymphopenia, and normal hepatic and kidney biology.

The lung CT-scan carried out on September 18 showed that less than 25% of the lung was impacted with patchy images.

#### Clinical case 2

A 61-year old woman in perfect health and receiving no treatment developed mild COVID-19 symptoms on September 28 (nasal obstruction, cough, fatigue, and headache) that disappeared on day 2 (Table 1). On October 1 (day 4), she had most of the COVID-19 symptoms including loss of taste, smell, and appetite. On day 6, she took 300 mg of broccoli capsules at 14h30. In 10 min, cough and nausea disappeared, and smell and taste improved. Fever decreased over 5 h from 38.3° to 37.2°. After 6 h, at 20h30, fever (38.2°), nasal obstruction and cough (VAS 5) reappeared. She took the same dose of broccoli with paracetamol (1000 mg) and the same improvements were noticed for a longer period of time (13 h). The third episode occurred the next morning. She then took broccoli capsules (300 mg) and the same sequence of improvement was observed for 8 h. The fourth episode occurred at 18h45 and, after taking broccoli (300 mg) and paracetamol (500 mg), the duration of the effect was around 15 h. The fifth episode occurred at 10h15. She took broccoli (300 mg), and symptoms did not re-occur until 19h15. She then took broccoli (300 mg) and paracetamol 500 mg and the same improvement was experienced for 14 h. Other episodes were less clear as she was improving. Interestingly, loss of smell and taste were improved by the broccoli, but the extent of this improvement may have required specific testing for a more accurate assessment (even though VAS is accurate).

**Table 1:** Clinical case 2

Day		Cough	Fever	Fatigue	Appetite	Smell loss	Taste loss	Nasal block.	Broccoli	Paracetamol
**4**		8	38.6	6	0	10	10	0		
**5**		8	38.5	10	5	10	10	5		
**6**	14h30	7	38.3	5	8	10	10	0	2	
	14h40	0	38	3	8	5	7	0		
	15h00	0	37.9	0	8	5	5	0		
	16h00	0	37.6	0	8	5	5	0		
	18h00	0	37.2	0	8	5	5	0		
**7**	20h30	5	38.2	5	8	5	5	5	2	1000 mg
	20h45	0	37.9	5	8	5	5	5		
	08h00	0	37.2	8	5	5	5	5		
	10h20	7	37.8	6	8	5	5	5	2	
	10h40	0	37.7	3	8	3	3	0		
	18h45	7	37.8	5	8	5	5	5	2	500 mg
	19h00	0	37.8	0	8	5	5	0		
**8**	10h15	7	37.2	0	5	5	5	5	2	
	10h30	0	37.2	0	5	3	5	3		
	14h00	2	37.2	0	4	4	4	0		
	16h30	3	37.3	0	3	3	3	0		
	19h15	7	37.2	0	3	3	3	0	2	500 mg
	19h30	0	37.2	0	3	3	3	0		
	21h00	0	37.2	0	3	3	3	0		
**9**	09h00	0	37.8	5	4	8	8	8	2	500 mg
	10h30	0	37.6	3	4	3	3	3		
	12h00	0	37.7	0	2	2	0	0		
	20h30	7	38	3	2	2	0	0	2	500 mg
	21h00	0	37.9	3	2	2	0	0		
	22h00	0	37.6	3	2	2	0	0		

The patient tested positive for SARS-CoV-2 on day 8.

#### Clinical case 3

A 63-year-old man with controlled hypertension and receiving losartan had the first COVID-19 symptoms on October 3. He had rhinorrhea, dry cough, incomplete loss of smell and taste, and fatigue on day 2. On day 3 at 11h00, he had severe cough and fever and took 300 mg of broccoli. Cough and nasal obstruction disappeared very rapidly but fever was slower (Table 2). He took the same treatment with 500 mg of paracetamol (after the third dose) five times, and cough (VAS 5–8) always disappeared within 10 min. Nasal obstruction showed a similar trend in case 3 (in case 2 there were no nasal symptoms). Loss of smell and taste (VAS 6) on the first 2 days were improved after the first broccoli capsule (VAS 5) and were overall largely improved (VAS 0 at the last evaluation of day 5). Fatigue and fever appeared to be improved by broccoli, but the results were less consistent. Paracetamol was given 5 times and increased the duration of the benefit obtained with broccoli.

**Table 2:** Clinical case 3

Day		Cough	Fever	Fatigue	Appetite	Smell loss	Taste loss	Nasal block.	Broccoli	Paracetamol
**1**		0	0	8	6	5	5	5		
**2**	18h00	5	37.2	0	0	6	6	0		
**3**	09h30	8	38	5	0	5	5	0		
	11h00	8	37.8	6	0	5	5	0	2	
	11h15	0	37.7	6	0	5	5	0		
	12h30	0	37.6	3	0	5	5	0		
	15h15	8	37	2	0	5	5	0		
	16h30	8	37.8	5	0	5	5	5		
	19h00	8	38.6	5	0	5	5	4	2	1000 mg
	19h15	0	39.2	7	0	5	5	0		
	21h00	0	38.8	9	5	5	5	0		
4	09h00	5	37.9	5	0	4	4	6	2	500 mg
	10h30	0	37.5	5	0	0	0	0		
	12h00	0	37	5	0	0	0	0		
	21h00	0	37.9	6	5	4	4	0	2	1000 mg
	21h15	0	37.7	0	5	4	4	0		
	22h00	0	37.4	0	5	4	4	0		
**5**	09h15	5	37.7	5	8	4	4	6	2	1000 mg
	09h30	5	38.1	5	5	4	4	0		
	12h00	3	37.6	3	0	3	3	0		
	12h30	0	37.2	0	0	0	0	0		
	15h30	5	37.8	0	0	0	0	3		
	17h00	0	38.6	7	0	0	0	3		
	21h00	4	37.9	3	0	0	0	3	2	1000 mg
	21h15	2	37.7	3	0	0	0	3		

The patient tested positive for SARS-CoV-2 on day 3.

### Cough provocation test in clinical case 1

The patient is allergic to grass pollen and cat, and has moderate-to-severe allergic rhinitis which is perfectly controlled by PRN medications. Further to the onset of COVID-19, he experienced mild to moderate nasal obstruction (VAS up to 6/10) and occasional episodes of mild rhinorrhea (up to 3/10). The rhinorrhea did not have the features of allergic rhinitis. The patient attempted to reduce nasal symptoms with azelastine-fluticasone propionate but this was not effective. The patient did not experience any sneezing, nasal pruritus, or eye symptoms. He proposed a score for his symptoms according to the VAS for allergic rhinitis (from 0 to 10).^20^, ^21^, ^22^ He noticed that nasal symptoms and cough always improved within minutes of ingesting the broccoli capsules and then reappeared with cough when the treatment became ineffective.

#### Design of the test

The patient also suffers from mild intermittent asthma, especially when exposed to high levels of air pollution. He uses formoterol-budesonide low dose when needed, with a maximal annual consumption of around 40 doses. He has a severe bronchial hyperreactivity. During the COVID-19 infection, he had symptoms — that were not well defined — in the form of an intermittent pruritus of the tracheobronchial tree, followed, within minutes, by dry cough. He had never experienced such symptoms. He used formoterol-budesonide low dose to reduce cough, but this was not effective. These symptoms were always followed by a more severe cough, and the patient ingested the broccoli capsules when cough was over 4 or 5/10 (VAS). There are many scores for cough ^23^^,^^24^ and he used a VAS score for “cough”. When he had pruritus, a deep inspiration followed by a rapid expiration led to immediate mild “wheezing” (not similar to asthma) and to cough that could be scored. He also used a score for induced cough during challenge, with some criteria. For spontaneous cough during challenge, he decided to count the number of coughs per episode (Table 4).

#### Open label induced cough challenge with broccoli (days 26–28 and 31–38)

In order to test the induced cough challenge and the dose that could be effective, the patient performed 3 induced cough challenges with a dose of 300 mg of broccoli (daily dose recommended). Similar to the FEV_1_, he took the deepest breath he could, and then exhaled as fast and as hard as possible and counted the number of coughs (see Table 2). He found that induced cough (by inhalation challenge) improved within 10 min after broccoli ingestion, and nasal obstruction even more rapidly. He then selected this dose for the DB, PC challenge.

After the DB, PC challenge, when the patient needed the broccoli capsules (VAS spontaneous cough >5/10), he performed an open challenge at least once a day. A total of 7 challenges were carried out (Table 3).

**Table 3:** Cough symptom score

Spontaneous cough	VAS	Induced cough challenge	score
≥5 coughs for one episode	3	persisting severe cough	9–10
2-4 coughs for one episode	2	>3 cough, intolerable	8
1 cough for one episode	1	>3 cough, severe	7
		>3 cough, mild	6
		2-3 cough severe	5
		2-3 cough mild	4
		1 cough	3
		Loud “wheezing”	2
		Some “wheezing”	1

**Table 4:** Open label induced cough challenge with broccoli (number of challenges: 10)

Time (min)	Mean score	SD	Minimum score	Maximum score
−5	7.5	0.53	7	8
Dosing	6.9	0.74	6	8
1	6.9	0.67	6	8
2	6.9	0.75	6	8
5	4.3	1.26	2	6
8	2.1	0.57	1	3
10	2.0	0.82	1	3
15	2.0	0.63	1	3
20	1.8	0.67	1	3
25	1.8	0.79	1	3
30	1.7	0.63	1	3
45	1.8	1.03	1	3
60	1.6	1.00	1	3

#### Double-blind induced cough challenge with broccoli (days 29–30)

A double-blind placebo-controlled trial was carried out on induced cough using 4 placebo capsules or 4 broccoli (300 mg) capsules. The challenge was performed double-blind to confirm that the effect of broccoli seed capsules was real and not biased. The placebo and broccoli capsules were prepared by the Pharmacie des Quatre Seigneurs (Montpellier). The capsules were made up of cellulose (opaque). Neither the broccoli nor the cellulose capsules had any taste, thus there was a total placebo effect. The investigator was not aware of the blinding, nor of the code that was broken after the test. Similar to the open challenge, he took the deepest breath he could, and then exhaled as fast and as hard as possible. He started the test when spontaneous cough VAS was over 5/10. He first measured spontaneous and induced cough scores as well as nasal VAS, 5 min before ingesting the capsule. He ingested the capsule and then measured the 3 parameters every minute for 10 min, at 12, 15, 17, 20, 25, 30, 45 and 60 min.

**Fig. 5:**
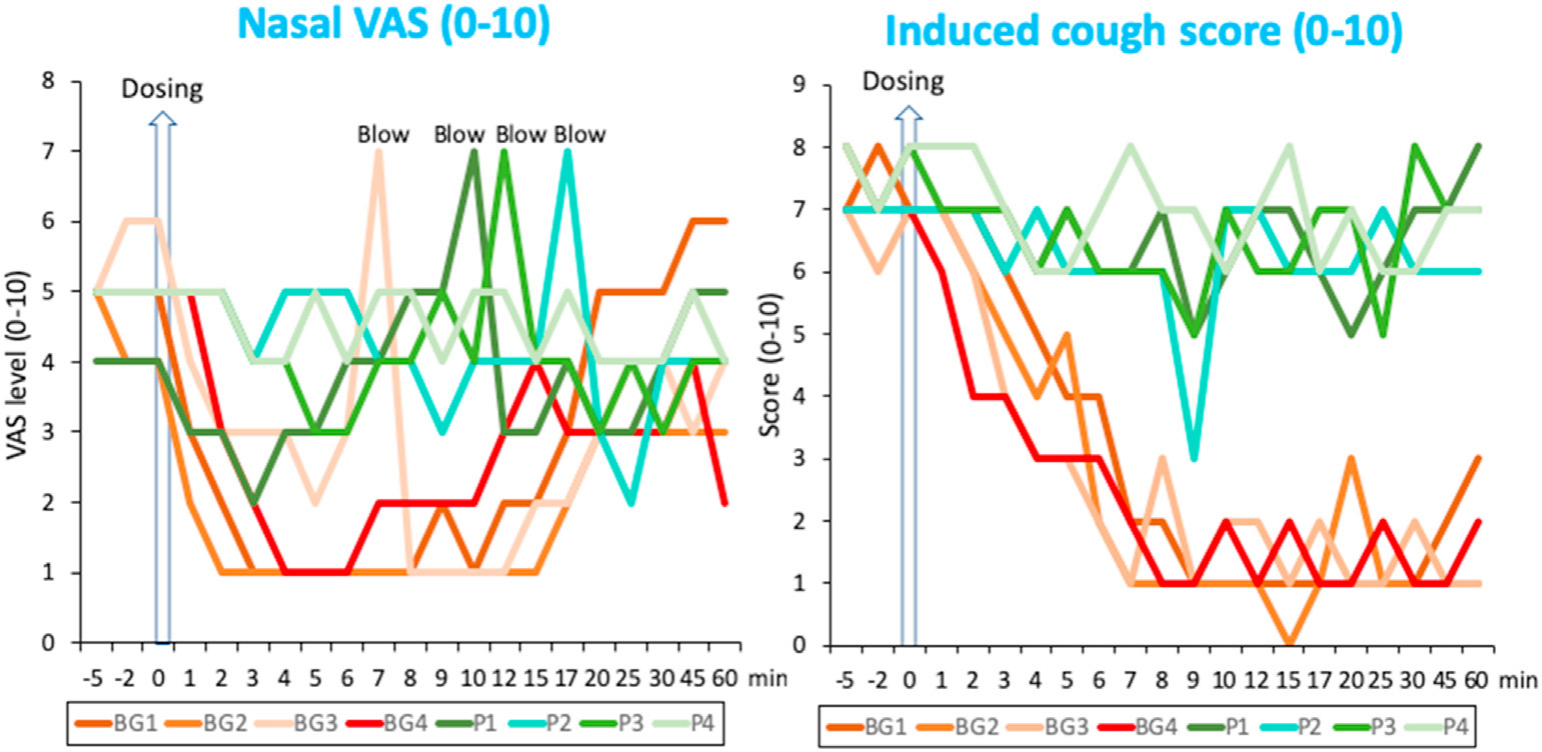
Double-blind placebo-controlled challenge (300 mg broccoli (BG) or placebo (P)) on spontaneous nasal and induced bronchial symptoms at days 29–30

In the broccoli group, induced cough score was around 7–8 before the dose and below 3 after 6–9 min (Fig. 5). Score levels remained at between 0 and 3 for the 60-min duration of the test. In the placebo group, there was merely no effect. The results for both the active and placebo groups were very consistent. The results for the active group were consistent with the open cough challenges (Table 3).

In the broccoli group, 6, 6, 10, and 10 episodes of spontaneous cough were recorded for over 60 min, compared to 21, 25, 26, and 28 in the placebo group.

In the broccoli group, nasal symptoms were reduced very rapidly (2–3 min), except for BG3. Nasal obstruction was the only symptom recorded during the baseline of BG1, BG2, and BG4, whereas both nasal obstruction and rhinorrhea (VAS: 3) were recorded during BG3 baseline. The patient needed to blow his nose at 7 min (VAS 7). After 12–15 min, symptoms increased. In the placebo group, symptoms were variable but were never below 2, and all 3 tests required nose blowing (indicated as VAS 7).

VAS spontaneous cough ≥5 was noted at 4h30, 5h00, 6h00 and 7h45 after the ingestion of the broccoli capsules, whereas it was present at 60 min (two cases), 1h15 and 1h30 after the ingestion of the placebo capsules.

## Discussion

These 3 experimental clinical cases, like any clinical case, should be considered carefully in terms of efficacy, and with caution in terms of safety. Moreover, in the first case, the patient and the investigator who proposed the treatment were the same person. The case has been well described and the study analyzed — as if it were to be published — right from the onset of symptoms and the first dose of the fast-acting broccoli capsules (day 3). Safety should be considered carefully as high doses are required.

Several foods act on Nrf2.^4^^,^^25^, ^26^, ^27^, ^28^, ^29^, ^30^, ^31^ It has been proposed but not confirmed that Nrf2-interacting nutrients may be effective in COVID 19.^32^, ^33^, ^34^, ^35^, ^36^ The cases presented herein are a proof-of-concept for the clinical effects of Nrf2-interacting nutrients in patients with COVID-19 using broccoli and glucoraphanin. We have proposed that differences in death rates between and within countries are associated with Nrf2-interacting nutrients.^1^, ^2^, ^3^ In the present study, there are 6 levels of evidence suggesting that broccoli components are effective in COVID-19: (i) During COVID-19, with initial symptoms (days 3 and 4) and the cytokine storm (days 7–12), a high dose of broccoli was always effective within minutes; (ii) When the broccoli was stopped, the same COVID-19 symptoms reappeared and were controlled with the same speed using the same treatment; (iii) Broccoli appeared to be effective within minutes on most of the COVID-19 symptoms except fever. However, for clinical case 2, smell and taste were reduced but specific tests would have been required for full appraisal;^37^ (iv) The effect of broccoli was also found in clinical case 1 during the recovery period after the cytokine storm; (v) A double-blind, placebo-controlled induced cough challenge was performed on days 28 and 29. The results were perfectly in line with 17 open induced cough challenges and with the clinical benefits observed on days 3, 4, 7, 8 (broccoli capsules only) and on days 9, 10, 11, 12 (broccoli capsules and paracetamol);

The label indicates that there is 10% of glucoraphanin in the broccoli capsules. However, the exact dosage could not be found, neither on the label nor on the website of the manufacturer. The capsule has therefore been analyzed and the doses of glucoraphanin are lower than those indicated on the label (3% instead of 10%). Glucoraphanin is an important component of broccoli because sulforaphane [1-isothiocyanato-4-(methylsulfinyl) butane], found in a stored form such as glucoraphanin in cruciferous vegetables, is the most potent natural activator of Nrf2.^38^ Present in the plant as its precursor, glucoraphanin, sulforaphane is formed through the action of myrosinase, a β-thioglucosidase present in either the plant tissue or the mammalian gut microbiome.^4^^,^^5^ Sulforaphane is a clinically relevant nutraceutical compound used for the prevention and treatment of chronic diseases and may be involved in ageing.^39^ However, the very early effects of broccoli cannot be attributed to sulforaphane.

The mechanisms of action of broccoli are unclear. The immediate effect on cough in COVID-19 was noted at 5–10 min after ingestion in both clinical cases. It is interesting to note that most COVID-19 symptoms are rapidly improved under broccoli, except cough which is either less well controlled or at a different kinetic (starts early and takes 2–3 h to be improved but not fully controlled). The speed of onset of broccoli is surprising but highly consistent (4 DB-PC induced cough challenges and 10 open label challenges), and it is possible that changes in oxidative stress can be very rapid. Nasal effects are even faster. Sulforaphane improves respiratory function for people with lung problems by increasing state 3 respiration and respiration linked to ATP synthesis in mitochondria.^40^, ^41^, ^42^, ^43^ As such, it also promotes glucose and lipid utilization. Thus, in particular, hypoxia-induced injuries are prevented.

Broccoli has an inconstant effect on asthma, 44, 45, 46 whereas sulforaphane from broccoli has a bronchoprotective response through Nrf2 ^47^ and reduces nasal response to diesel exhaust particulates.^26^ Sulforaphane has an antibiotic function^48^ and antiviral functions,^49^ as well as antiproliferative activities that lead to its use as a chemopreventive agent.^50^^,^^51^ Oxidant activation of the airway neurons induces respiratory depression, nasal obstruction, sneezing, and cough.^52^^,^^53^

However, other mechanisms may also be proposed. The cough reflex is regulated by vagal, sensory afferent nerves which innervate the airway. The transient receptor potential (TRP) family of ion channels is expressed on sensory nerve terminals, and, when activated, can evoke cough.^54^ Diesel exhaust can act on airway sensory neurons.^55^ TRPA1 (Transient Receptor Potential Ankyrin type 1), a member of the TRP gene family, is a major oxidant sensor in airway sensory neurons.^56^^,^^57^ It is possible that the early effects on the nose may be mediated through these mechanisms through a TRPA1 reflex. Another mechanism may be proposed. The TRPA1 channel may be activated and desensitized at high doses within seconds by electrophilic pungent compounds.^58^ Broccoli contains flavonoids ^59^^,^^60^ including quercetin,^59^ naringenin, ^61^ and kaempferol ^62^ that interact with TRPA1 and TRPV1 (Transient Receptor Potential Vanillin type 1). We suggest that electrophilic ligands activate and desensitize TRPA1 and that they also activate Nrf2, thus blocking the activation of TRPA1 by reactive oxygen species (ROS) produced by COVID-19.

Paracetamol (acetaminophen) has TRPA1-independent antipyretic effects ^63^ and TRPA1-dependent effects on pain.^64^ The electrophilic metabolites N-acetyl-*p*-benzoquinone imine (NAPQI, hepatotoxic metabolite) and *p*-benzoquinone, but not paracetamol itself, activate TRPA1.^65^ They also activate and sensitize TRPV1 (transient receptor potential vanillin 1) by interacting with intracellular cysteines.^66^^,^^67^ NAPQI also directly activates Nrf2,^68^ and benzoquinone desensitizes TRPA1.^69^ TRPA1, an excitatory ion channel originally associated with the receptor of mustard oil in sensory neurons,^70^ plays a pivotal role in detecting cysteine-reactive irritants and in augmenting sensory or vagal nerve discharges to evoke several COVID-19 symptoms including cough.^54^^,^^71^^,^^72^ TRPV1, also known as the capsaicin receptor^73^, is also involved in cough and lung injury. TRPA1 and TRPV1 are probably desensitized and have a cross-talk with Nrf2 (Bousquet et al, paper submitted, available online).^74^

Medications in capsules can be active after 1–2 min and pass into the blood stream within 2 min. The effects on cough are probably more complex. The early effects of the ingestion of the broccoli capsules can only be ascribed to broccoli, since sulforaphane cannot be produced so fast. The rapid but steady decrease in cough symptoms may be associated with the same mechanisms, but probably include other mechanisms. It is unlikely that cough in COVID-19 is associated mainly to eosinophilic inflammation, since inhaled corticosteroids appeared to be ineffective in the first clinical case.

One question that cannot be answered is whether broccoli reduced the severity of the cytokine storm. The patient of the first clinical case was at high risk of developing severe symptoms during the cytokine storm due to the number of symptoms he experienced during the first phase of COVID-19 as well as his age and sex. Moreover, he developed an early cytokine storm on day 6 that was long lasting. However, symptoms were controlled by the treatment and he never suffered from dyspnea or from a respiratory rate of above 24. He had a mild inflammatory response and the CT-scan showed few COVID-19 typical lesions. It may be suggested that broccoli prevented a severe COVID-19 illness. The second clinical case was carried out during the cytokine storm and symptoms were improved by a lower dose of broccoli.

Using the dose of broccoli recommended by the manufacturer, there was no preventive effect on SARS-CoV-2. However, it is likely that the dose was insufficient for any such effect. The doses required for effective action during the COVID-19 symptoms of case 1 were quite high and should raise safety concerns. We used a daily dose of under 200 μmol of glucoraphanin, and trials with doses of up to 800 μmol daily have been reported without safety concerns.^9^^,^^25^^,^^75^ However, such a high dose may have pharmacologic effects such as the antagonism of aryl hydrocarbon receptors modulating the CYP1 (Cytochrome P450, family 1, subfamily A, polypeptide 1) family of cytochromes P450 ^76^ or a direct effect of CYP1.^77^ These effects must be studied carefully before any trial is carried out. Moreover, in broccoli seeds, there are many other compounds that may have pharmacologic properties when ingested at high doses.^25^ Thus, this experimental clinical case is a proof-of-concept to confirm the hypothesis but cannot be used in practice before more safety data are available. Another safety issue is the reduction of the cough that may have been helpful in expelling pathogens. This would be of interest in the first week of COVID-19 as the cytokine storm and the recovery phase progress without infection.

The patient of the first clinical case is allergic to grass pollen and cat and also suffers from mild intermittent asthma. It might be possible that some of the effects of the Nrf2-interacting nutrients are related to bronchial hyperreactivity, but inhalation of formoterol-budesonide low dose did not reduce cough. However, it cannot be ruled out that the results observed on cough may be partly associated with asthma or allergy. The patient also attempted to reduce nasal symptoms with azelastine-fluticasone propionate but there was no effect. Moreover, nasal symptoms are mainly characterized by nasal obstruction alone that is not associated with allergic triggers.

These 3 clinical cases should be considered with care and confirmed by proper trials on efficacy and safety. However, they are in keeping with the hypothesis that cabbage may reduce COVID-19 ^1-3^ and with many papers highlighting the role of nutrition in COVID-19. What is novel is the demonstration in all 3 clinical cases that the hypothesis is supported and that the speed of action of the nutrients is so fast. Research is needed to (i) confirm the case, (ii) investigate in which patients the treatment is safe and effective, (iii) differentiate the effects on Nrf2 and TRPA1, (iv) assess the different broccoli compounds that are active (early and late), (v) assess the dose required for effective action, (vi) confirm the speed of onset of action, and (vii) optimize the nutrients to be administered (as nutrients or active molecules).

## Funding

None.

## Availability of data and materials

Not applicable.

## Authors' contributions

JB proposed the treatment and was the patient. He discussed it with JMA, WC and TZ. JB wrote the paper. VLM, HB and JR were the physicians of JB. AB, JPC, AAC, AF, TH, GI, LK, PK, EM, JM, BS and AV were the ARIA reviewers. RdlT and NPL discussed the broccoli content. All authors were requested to comment on the concept and to review the paper.

## Ethics approval and consent to participate

Not applicable since this was a food supplement used at regular doses by the patients of cases 2 and 3 and at higher doses (case 1) by the author of the study.

## Consent for publication

All authors gave their informed consent to publish the work.

## Declaration of competing interest

None of the authors declared any competing interest.
